# Concurrent Incidental Presentation of Primary Retroperitoneal Follicular Lymphoma With Acute Pancreatitis

**DOI:** 10.7759/cureus.11687

**Published:** 2020-11-24

**Authors:** Dominic Gaziano, Aelia Akbar, Komal Lakhani, Toochukwu L Okafor, Siddharth Bhesania

**Affiliations:** 1 Internal Medicine, Amita Health, Chicago, USA; 2 Public Health, Loyola University Medical Center, Chicago, USA; 3 Internal Medicine, Lenox Hill Hospital, New York, USA; 4 Internal Medicine, Larkin Community Hospital, Hialeah, USA; 5 Internal Medicine, New York-Presbyterian Brooklyn Methodist Hospital, New York, USA

**Keywords:** follicular lymphoma, endoscopic ultrasound (eus), acute pancreatitis, large b cell lymphomas, non-hodgkin’s lymphomas, laparoscopic cholecystectomy

## Abstract

Background: Follicular lymphomas are a common type of non-Hodgkin’s lymphomas (NHL). Presentation varies widely from being asymptomatic to painless peripheral lymphadenopathy to classic B symptoms. We present an unusual case of follicular lymphoma where the patient initially presented with signs and symptoms of acute pancreatitis. The aim of this study is to recognize the challenges faced while diagnosing retroperitoneal NHL and the need for timely management of this disease.

Case report: A 66-year-old Hispanic female with a medical history of treatment compliant asthma and hypertension presented to the ER with complaints of abdominal pain in the right upper quadrant with serum lipase >3000 U/L and elevated liver function tests (LFTs), aspartate aminotransferase (AST) 139 U/L, alanine aminotransferase (ALT) 65 U/L, alkaline phosphatase (ALP) 122 U/L. Abdominal ultrasound identified gall bladder wall thickening and dilation of biliary ducts. CT scan showed soft tissue mass in the retroperitoneum, measuring 9.3x4.8cm which wrapped around the aorta and pushed it off the spine. After two days of conservative management, her pain resolved and lipase levels normalized, she was discharged and scheduled for outpatient endoscopic ultrasound (EUS) with biopsy of the retroperitoneal mass. The next day, the patient presented to the ER with similar pain, and labs again showed elevated lipase, EUS, and fine needle biopsy of mass showed CD-10 positive B-cell lymphoma. The patient was discharged after the resolution of pain. A positron emission tomography (PET) scan four weeks after the initial CT scan showed an increase in tumor size without any metastatic lesions. While awaiting core biopsy, the patient presented to the ER for the third time with worsening abdominal pain, lipase >3000 IU/L, and ultrasound showing cholelithiasis with cholecystitis. The patient underwent laparoscopic cholecystectomy. Core needle biopsy of paraspinal lymph nodes showed grade 1-2 follicular lymphoma. Finally, the patient underwent six cycles of chemotherapy with Bendamustine and Rituximab and after the fourth cycle, a repeat CT scan showed resolution of adenopathy with minimal residual soft tissue attenuation in retroperitoneum.

Discussion: NHL rarely occurs in retroperitoneum and its diagnosis is challenging. Our patient presented with the primary and unique occurrence of follicular lymphoma in the retroperitoneum. She presented with symptoms of an acute abdomen with elevated lipase and LFTs. She underwent multiple hospitalization and cholecystectomy before the correct diagnosis was made and until she was treated for follicular lymphoma.

Conclusion: This study emphasizes the importance of being vigilant when a patient presents with unusual presentations of a disease in order to diagnose and treat the condition early to decrease the risk of complications and to mitigate the risk of poor outcomes.

## Introduction

Presentation and histology of non-Hodgkin lymphoma (NHL) vary widely which makes the diagnosis challenging. Their timely diagnosis is necessary as effective therapy is available for many subtypes. Follicular lymphomas are the second most common histological subtypes of NHL after the diffuse large B cell lymphomas (DLBCL). There has been a significant rise in the incidence of NHL over the past few decades including the subtypes of DLBCL and follicular lymphomas, irrespective of the HIV [1,2]. According to the National Cancer Institute (NCI) SEER (Surveillance, Epidemiology, and End Results) Program, NHL comprises 4.3% of all the new cancer cases with an estimated 77,240 new cases of NHL in the year 2020 [3]. NHL affects people of all ages, races, and socioeconomic statuses. They can be found anywhere in the lymphatic system of the body, therefore, the clinical presentation varies widely, depending on the histologic subtype and site of involvement. NHL commonly presents as enlarging and non-tender lymphadenopathy associated with symptoms of immunodeficiency or uncommonly with symptoms of obstruction of GI or respiratory tracts. In two-thirds of the cases, NHL occurs in peripheral lymph nodes and in one-third in extranodal sites such as in Waldeyer’s ring, stomach, and gastrointestinal tract. Rarely, NHL occurs in the retroperitoneum where it is difficult to diagnose due to the location as well as non-specific symptoms at presentation [4-6].

We report a case of primary retroperitoneal non-Hodgkin follicular lymphoma which presented as acute pancreatitis and the challenges faced while diagnosing retroperitoneal NHL.

## Case presentation

A 66-year-old Hispanic female with a medical history of asthma and hypertension presented to the Emergency Department with complaints of abdominal pain of score 7/10 and associated nausea. The pain was sharp, constant, in the right upper quadrant, and radiated to the back. She reported no fever, night sweats, vomiting, diarrhea, or constipation. The patient had experienced similar intermittent pain with the intake of fatty foods over the past one month. She was an ex-smoker and had quit smoking 14 years ago. She denied any alcohol intake. She underwent no previous surgeries other than tubal ligation.

Physical examination revealed a thin woman who was moaning and groaning in pain, with normal skin color and no generalized lymphadenopathy. Vital signs were normal. There was tenderness to deep palpation in the right upper quadrant on the abdominal examination, and there was no palpable abdominal mass. The rest of the examination was unremarkable.

Laboratory workup yielded a lipase level >3000 U/L and elevated liver function tests (LFTs) with aspartate aminotransferase (AST) 139 U/L, alanine aminotransferase (ALT) 65 U/L, alkaline phosphatase (ALP) 122 U/L. Abdominal ultrasound identified gallbladder wall thickening with pericholecystic fluid. A diagnosis of cholecystitis and pancreatitis was being considered. 

**Figure 1:**
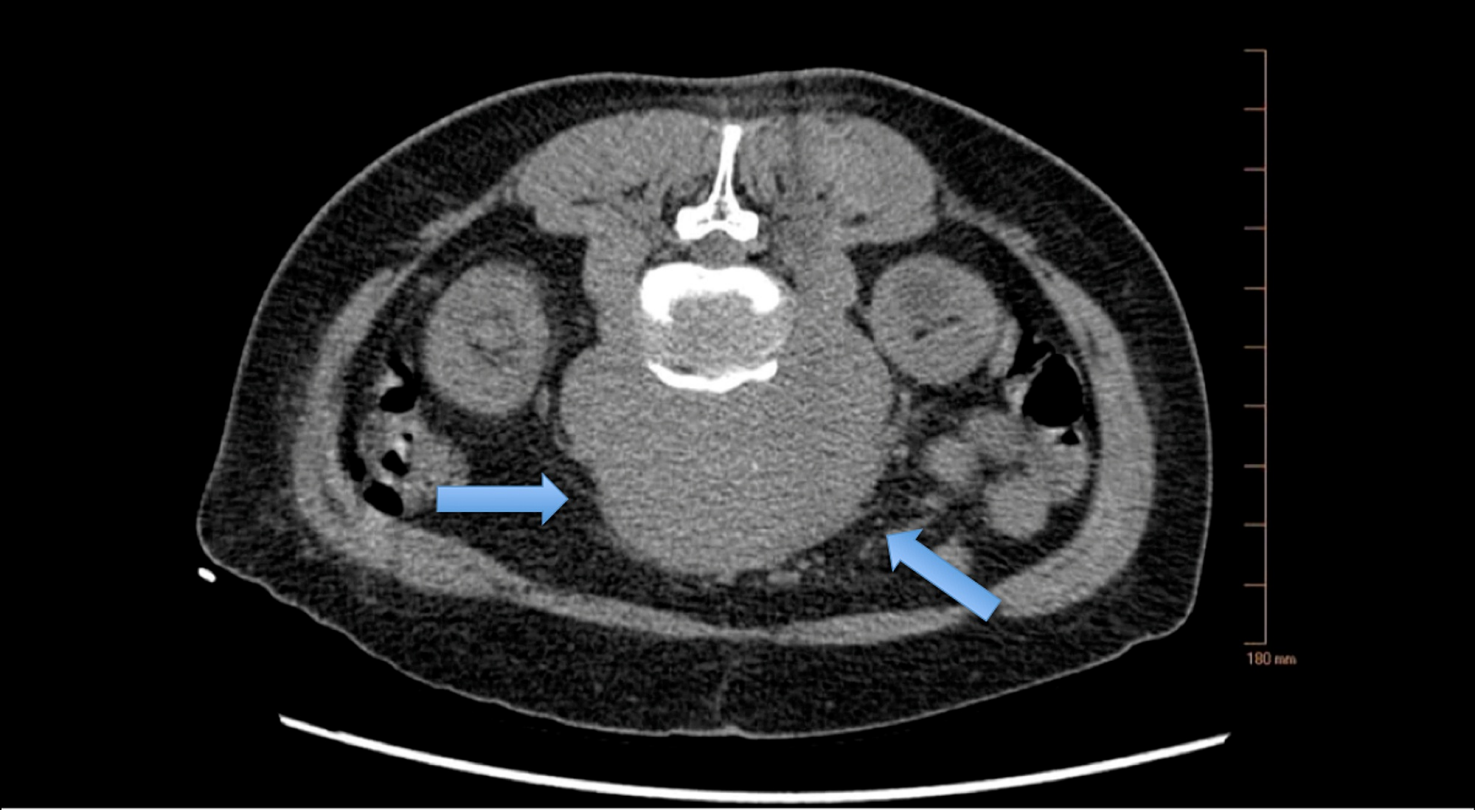
CT scan shows the large retroperitoneal mass.

The CT scan showed a large amount of soft tissue mass in the retroperitoneum measuring 9.3 x 4.8 cm. The mass wrapped around the aorta and pushed it off the spine (Figure 1). It forced the uncinate process of the pancreas and bowel anteriorly and displaced the inferior vena cava. A minimal amount of induration was seen around the pancreas related to the retroperitoneal mass or pancreatitis. 

**Figure 2:**
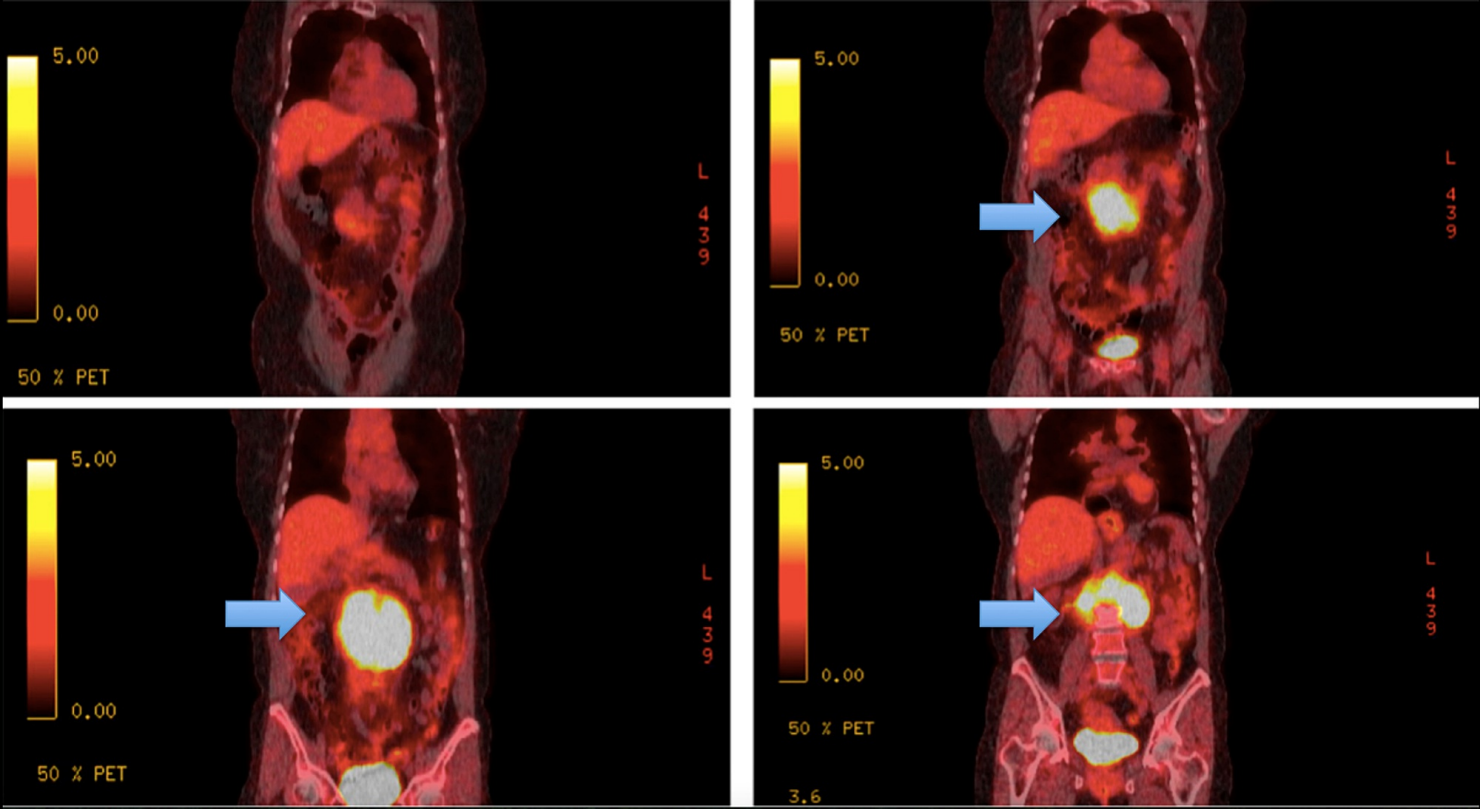
Positron emission tomography (PET) scan shows increase uptake in mass suggesting malignant mass.

After conservative treatment, the patient’s pain resolved, and lipase levels normalized, and she was discharged to follow up for outpatient endoscopic retrograde cholangiopancreatography (ERCP) and endoscopic ultrasound (EUS) with biopsy of the retroperitoneal mass. One day later, the patient presented to the ER with similar abdominal pain, and laboratory workup during admission showed elevated lipase levels and normal LFTs. After resolving the lipase level's pain and normalization, the patient later underwent EUS, which confirmed the previous CT scan findings. The fine-needle biopsy of the mass showed a CD-10 positive, CD-5 negative B-cell lymphoproliferative disorder on flow cytometry. The positron emission tomography (PET) scan performed four weeks after the initial CT scan showed the tumor's size slightly larger than the previous one, with a transverse diameter of 10.1 x 5.9 cm and a cephalocaudal length of 9.5 cm without any metastatic lesions (Figure 2).

While awaiting the core biopsy and chemotherapy workup, the patient presented to the ER for worsening abdominal pain for the third time. With lipase levels >3000 U/L and ultrasound showing cholelithiasis with cholecystitis, the patient underwent laparoscopic cholecystectomy. To confirm and classify B cell lymphoma type, a core needle biopsy of the paraspinal lymph node was done. It was consistent with grade 1-2 follicular lymphoma.

The patient underwent six cycles of bendamustine and rituximab treatment without any side effects. After four cycles of chemotherapy, a CT scan was performed to determine the response to therapy. CT scan showed resolution of lymphadenopathy with minimal residual soft tissue attenuation in the retroperitoneum.

## Discussion

Malignant lymphomas are divided into two broad categories - Hodgkin’s lymphomas and non-Hodgkin's lymphomas (NHL). NHL is the most common type of lymphoma and comprises about 90% of the cases [1]. At presentation, the NHL may be indolent or aggressive. The most common subtype of NHL is diffuse large B-cell lymphomas (DLBCL) followed by follicular lymphomas which comprise approximately 20% of the total NHL cases [7]. Follicular lymphomas are usually indolent at the time of diagnosis. The median age at diagnosis for follicular lymphomas is 63 years, with the rate of new cases is 2.9 per 100,000 in males compared to 2.5 per 100,000 in females [1,7,8]. NHL commonly occurs in the retroperitoneum compared to Hodgkin’s lymphomas and presents with varying clinical features [9]. Our patient presented with signs and symptoms of acute pancreatitis at the initial presentation which is, according to our knowledge, never reported before as an initial presentation of a primary follicular lymphoma [4-6,9].

Retroperitoneal masses are diagnostically challenging due to their location and wide range of differential diagnoses including solid versus cystic and neoplastic versus benign lesions. Lymphomas comprise 33% of all primary retroperitoneal mass [10]. Presentation varies from abdominal pain or discomfort to palpable abdominal mass or asymptomatic. CT abdomen and pelvis are done to determine the exact location and extent of mass including size and spread which helps in the diagnosis and staging of lymphoma. Our patient underwent a CT scan at her first visit, showing retroperitoneal mass. The patient was treated conventionally and sent home to do a follow-up after ERCP and EUS with biopsy. There were two subsequent ER visits and hospitalizations for similar symptoms and cholecystectomy before we could diagnose that her retroperitoneal mass was follicular lymphoma. After doing fine needle aspirations (FNA) with paraspinal lymph node biopsy, which was suggestive of follicular lymphoma, the diagnosis was confirmed with flow cytometry. Though excisional biopsy is the gold standard for the diagnosis of lymphoma, FNA of the mass or involved lymph node is a less invasive and time-saving procedure [9].

Treatment for follicular lymphoma includes radiation therapy, chemotherapy, and stem cell transplant especially in younger patients with relapsing disease. Multiagent chemotherapy options are rituximab, cyclophosphamide, vincristine, prednisone (R-CVP); rituximab, cyclophosphamide, doxorubicin, vincristine, prednisone (R-CHOP); CHOP immediately followed by tositumomab/iodine-131 radioimmunotherapy (CHOP-RIT) bendamustine plus rituximab [11], rituximab plus lenalidomide (non-chemotherapeutic regimen). Our patient underwent treatment with six cycles of bendamustine plus rituximab and a CT scan after four cycles of chemotherapy suggested resolution of the mass with the minimal residual disease [4].

## Conclusions

Follicular lymphoma has a very good overall prognosis of a relative five-year survival rate if diagnosed and treated early in the course of the disease. We emphasize on diagnosing and treating these patients on time to reduce morbidity and mortality associated with it. 
